# EHMTI-0390. The attitude of migraineurs to alternative therapies

**DOI:** 10.1186/1129-2377-15-S1-M4

**Published:** 2014-09-18

**Authors:** E Csepany, M Toth, D Janoska, Z Nagy, E Balogh, T Gyüre, G Bozsik, C Ertsey

**Affiliations:** 1 János Szentágothai Neurosciences PhD School, Semmelweis University, Budapest, Hungary; 2 Headache Service, Vaszary Kolos Hospital, Esztergom, Hungary; 3 Faculty of Medicine, Semmelweis University, Budapest, Hungary; 4 János Szentágothai Neurosciences PhD School, Semmelweis University, Budapest, Hungary; 5 Department of Neurology, Semmelweis University, Budapest, Hungary

## Introduction

The growing interest in alternative therapies (ALTs) is a worldwide phenomenon. Headache and migraine are among the top five conditions treated with such therapies, although scientific proof of efficacy is often lacking.

## Objective

To explore the attitude of Hungarian migraineurs to alternative therapies.

## Methods

Questionnaire survey conducted at two headache centres. A willingness to pay approach was used to assess the importance of alternative therapies for the patients.

## Results

75 patients (mean age: 35.4±12.4 years; 70 women) were enrolled. Thirty-four (44%) had already tried at least one ALT. Ninety percent of the patients were willing to pay for ALTs recommended for their migraines, on average 19% of the Hungarian monthly net minimum wage. If scientific evidence of efficacy was lacking, 57% of the patients would pay for the ALTs (on average 14% of the net minimum wage). If the ALTs were associated with side effects, only 33% of patients would try them. Actual use and willingness to use was higher among patients with higher levels of education. If two therapies were equally effective, 65% would choose an ALT and 11% would prefer the conventional therapy (24% having no preference). If the treatments’ side effects were equal, 36% would choose the ALT and 22% the conventional treatment (p<0.001 for the differences between the two scenarios).

## Discussion

The majority of migraineurs had positive attitudes towards ALTs. This is probably due to ALTs being considered safer than conventional therapies. Patients with higher levels of education have a higher preference for ALTs.

No conflict of interest.

